# How practical are the “teaching reforms” without “curricular reforms”?

**DOI:** 10.4103/0975-9476.72612

**Published:** 2010

**Authors:** Kishor Patwardhan

**Affiliations:** *Department of Kriya Sharir, Faculty of Ayurveda, Institute of Medical Sciences, Banaras Hindu University, Varanasi - 221 005, India. E-mail: patwardhan.kishor@gmail.com*

Sir,

This letter is in response to the article titled “Teaching reforms required for Ayurveda” by J. Narayan,[1] the Vice President, Central Council of Indian Medicine (CCIM).

Although the intentions of the author in the said article seem to be genuine and highly relevant, most of the issues that he has raised in connection with the weaknesses in the existing teaching-learning process should be looked at from a wider perspective. Below, I have tried to explain my concerns.

## “Teaching reforms” without the “curricular reforms”?

J. Narayan seems to be trying to suggest teaching reforms in the absence of curricular reforms as he seems to be content with the new curriculum that has been developed by CCIM. Well, one must remember that the quality of training that is imparted during a postgraduate program becomes vital in giving shape to a future teacher, and surprisingly, the postgraduate curriculum of almost no subject states that it aims at producing capable teachers.[2] Similarly, when J. Narayan notices the absence of several good practices like *Tadvidya Sambhasha* (participating in colloquia) and “problem-based learning and teaching” among the teachers, doesn’t he feel the necessity of introducing the students to certain important domains like clinical decision making, ethical decision making, using clinical record-keeping software, carrying out reasonably refined searches of medical information databases over the Internet, and using telemedicine, during their postgraduate years of study? Hasn’t the purview of *Tadvidya Sambhasha* crossed the barriers of a seminar hall in the present era of Internet?

## Problems in the newly framed syllabus

Although the newly introduced syllabus is far better than the existing one, it still has many discrepancies. These discrepancies are mainly because of the fact that most of our teachers wrongly regard the “syllabus” as equivalent to the “curriculum.” Actually, “curriculum” refers to the totality of content to be taught and the skills a student is expected to develop during the entire program. Thus, a curriculum subsumes a syllabus. The following are the major concerns related to the new curriculum that need attention:

- First, there are no appreciable differences between the contents of graduate and postgraduate levels of the syllabi in many of the subjects.
- Second, the entire syllabus needs to be classified on the basis of “must know,” “desirable to know,” and “nice to know” categories. This pattern is followed by many universities in India, and as an example, one can consult the MBBS (Bachelor of Medicine and Bachelor of Surgery) syllabus framed by MUHS (Maharashtra University of Health Sciences).[3] Unless this is done, the student will not know which topics in the syllabus are more important/applicable and which are less. For instance, descriptions related to the enumeration of anatomical structures and enumeration of different diseases as per different *Samhitas* (classical Ayurveda textbooks) must be included under “nice to know” category as these have least clinical applicability.
- Third, the syllabus does not specify anything about the pattern of questions that will be asked during examinations. It should be mandatory to frame at least 60–65% of examination questions from the “must know” category.
- Fourth, the syllabi of most subjects do not speak about the training that should be given in teaching methodology. Ideally, at least a few weeks of time during the postgraduate program in each area of specialization should be dedicated to developing skills in teaching methods in the form of using audiovisual aids, preparing lectures, delivering seminars, using e-content for teaching, etc.

## NET-like eligibility test is required

Unlike other streams of higher education, no National Eligibility Test (NET) like mechanism exists in Ayurveda[4] at present and every individual having just passed a postgraduate level of examination is eligible to enter the teaching profession. This is the reason why training in teaching methods has to be incorporated in postgraduate programs of Ayurveda. Even during interviews to select candidates for teaching posts, lecturing skills are generally not assessed.

## Results of a nationwide survey

Our team completed a nationwide survey on Ayurveda education in 2008 that included interns, postgraduate students and teachers from more than 30 Ayurveda institutions spread across 18 states of India. The study included 644 students and 378 teachers and we have reported our findings in two research papers in two international journals.[5,6]

Our study indicates that most students are not satisfied with their training in particular areas like *Panchakarma* (five basic purification therapies), *Kshara Sutra* (medicated thread used in the treatment of anal fistula and hemorrhoids), and *Jalaukavacharana* (leech therapy) at graduate level.[5] In addition, our study also showed that graduates are not trained sufficiently in handling clinical emergencies at primary healthcare level through Ayurveda. In general, we concluded that the exposure to basic clinical skills is insufficient during graduate programs.[5]

## Why new programs?

In addition to the above-mentioned findings, we have also noted that there is a considerable level of career-related anxiety among students because of limited employability.[6] Considering this, I do not understand the rationale behind starting another postgraduate diploma level of education, and also a 7-year graduate level program. Given the limited varieties of patients visiting hospitals in Ayurveda colleges,[5] will this not affect the quality of training in already existing programs?

To summarize, what I am emphasizing here is that there is no point in suggesting teaching reforms when the actual need is for curricular reforms. However, I am not in favor of simply adding many more points to the existing syllabus; rather, I suggest that possibilities like recruiting some subject experts other than Ayurveda teachers should also be explored.
